# Assessing the Risk of Nodal Metastases in Canine Integumentary Mast Cell Tumors: Is Sentinel Lymph Node Biopsy Always Necessary?

**DOI:** 10.3390/ani11082373

**Published:** 2021-08-11

**Authors:** Roberta Ferrari, Patrizia Boracchi, Lavinia Elena Chiti, Martina Manfredi, Chiara Giudice, Donatella De Zani, Carlotta Spediacci, Camilla Recordati, Valeria Grieco, Elisa Maria Gariboldi, Damiano Stefanello

**Affiliations:** 1 Dipartimento di Medicina Veterinaria, Università degli Studi di Milano, 26900 Lodi, Italy; lavinia.chiti@unimi.it (L.E.C.); martina.manfredi@unimi.it (M.M.); chiara.giudice@unimi.it (C.G.); carlotta.spediacci@unimi.it (C.S.); camilla.recordati@unimi.it (C.R.); valeria.grieco@unimi.it (V.G.); damiano.stefanello@unimi.it (D.S.); 2 Dipartimento di Scienze Cliniche e di Comunità, Laboratorio di Statistica Medica, Biometria ed Eopidemiologia “G.A. Maccacaro”, Università degli Studi di Milano, 20122 Milano, Italy; patrizia.boracchi@unimi.it; 3 Centro Clinico–Veterinario e Zootecnico–Sperimentale, Veterinary Teaching Hospital, Università degli Studi di Milano, 26900 Lodi, Italy; donatella.dezani@unimi.it (D.D.Z.); ElisaMaria.Gariboldi@guest.unimi.it (E.M.G.)

**Keywords:** mast cell tumor, sentinel lymph node, metastasis, staging, lymphoscintigraphy

## Abstract

**Simple Summary:**

Mast cell tumor (MCT) is the most common integumentary neoplasm in dogs. The draining lymph node (LN) is the first site of histologically detectable metastasis, even if not clinically altered. The recent literature showed the discrepancy between the regional LN and the truly draining LN (the so-called sentinel lymph node—SLN) in dogs with integumentary MCT. However, the mapping and biopsy of SLN include additional anesthesiologic, diagnostic and surgical procedures, and additional cost. The study aimed to assess the possible association between clinicopathological variables and SLN status. Sixty-six cases of low-grade cutaneous and subcutaneous MCT in dogs admitted to the mapping and biopsy of SLN were included. An MCT dimension smaller than 3 cm seems to correlate less with occult or overt SLN metastasis, although further study should confirm the exclusion of cases with this variable to the mapping and biopsy of the SLN. In contrast, the high association of tumor ≥ 3 cm and subcutaneous MCT with overt SLN metastasis involves a solid suggestion for those procedures in the presence of one of these variables.

**Abstract:**

The recent literature supports the sentinel lymph node (SLN) biopsy in dogs with MCT due to discrepancy with the regional lymph node and the high percentage of occult metastasis. However, the SLN biopsy includes additional anesthesiologic, diagnostic, and surgical procedures, and additional costs. The study aimed to assess the association between clinicopathological variables and SLN status, determining the identification of dogs at lower risk of SLN metastases. Dogs with integumentary MCT were admitted to the lymphoscintigraphic mapping and subsequent biopsy of SLN. The association between clinicopathological variables of MCT and SLN status was statistically tested, both considering occult and overt metastasis together (HN2-HN3) and overt metastasis (HN3) alone. Fifty low-grade cutaneous MCT and 16 subcutaneous MCT were included. A small to moderate association between integumentary MCT ≥ 3 cm and HN2-HN3 SLN was found. A strong association of integumentary MCT dimension and subcutaneous MCT with HN3 SLN occurred. Dimension of low-grade cutaneous and subcutaneous MCT seems to correlate with SLN status, but additional study should confirm this data before excluding small MCT to the SLN biopsy. On the contrary, the study results induce a solid suggestion for mapping and biopsy of the SLN in MCT > 3 cm and subcutaneous MCT.

## 1. Introduction

The identification of nodal metastasis in canine mast cell tumor (MCT) has undergone several changes during the last decade. Initially, regional lymph node (RLN) biopsy was suggested as the standard of care in dogs undergoing excision of integumentary mast cell tumor, given the low sensitivity and specificity of lymph node cytological sampling [1,2,3,4]. The attention for lymph node assessment was further supported by the observation of early and overt histological nodal metastases even in non-palpable/normal sized lymph nodes and in low-grade MCT [3,4,5,6]. 

More recently, the changes in nodal evaluation have led to questioning which lymph node should be assessed in the absence of clinically evident pre-operative lymph node alterations. Indeed, different mapping procedures have been applied to identify the sentinel lymph node (SLN) in dogs with cutaneous and subcutaneous MCT [4,5,6,7]. The disagreement between the anatomically closest RLNs and the actual draining SLNs is a common result: 42% of dogs had SLNs differing from the RLN in Worley’s study (2014); SLNs did not correspond to the RLN in 63% of cases in the study by Ferrari et al. (2020); clinicians would have incorrectly predicted the draining lymph node in 45.8% of MCT included in the a recent study by Fournier et al. (2020); 28% of SLN differed from the RLN in the Lapsley et al. study (2021). Moreover, a high variability in the number of SLNs excised in each dog has been consistently reported [4,5,6,7]. These results highlight the importance of SLN mapping and biopsy to achieve an accurate and personalized nodal staging and undermine the prognostic results reported in the previous studies focused on RLN evaluation. Conversely, SLN mapping and biopsy include additional anesthetics, diagnostic and surgical procedures, and additional costs for the owner, although to date there are no data available on the clinical benefits of these procedures, especially in case of non-high metastatic risk MCT [8,9,10]. Furthermore, information is lacking on the potential morbidity related to the removal of non-metastatic SLN.

Hence, the present study aims to explore the association between clinicopathological features and SLN metastases in dogs with integumentary MCT in order to determine if it is possible to correctly identify dogs at lower risk of SLN metastases. We hypothesize that no prognostic factors could help in predicting the risk of SLN metastasis and that surgical biopsy of the SLN should always be suggested. 

## 2. Materials and Methods

### 2.1. Sample Population

Client-owned dogs with cytologically confirmed integumentary MCT undergoing curative intent surgical resection of the tumor and SLN(s) were prospectively included from January 2017 to December 2020. To be eligible for SLN mapping, dogs must have staged negative for distant metastasis at preoperative ultrasound-guided spleen and liver cytology and have no clinical/ultrasonographic evidence of RLN metastases (based on Suami et al., 2013) [11]. This study did not include experimental animals and it was performed on client-owned dogs affected by integumentary mast cell tumor referred to our institutions for therapeutic purpose. All owners gave us consent for staging and treatment procedure, as well for data recording.

### 2.2. Sentinel Lymph Node Mapping, Biopsy, and Histological Evaluation

The SLN was identified by pre-operative lymphoscintigraphy and excised under intraoperative gamma-probe guidance as previously described [6,7,12]. Briefly, planar lymphoscintigraphy was performed the day before or the same day of surgery and consisted of a peritumoral injection of Technetium-99m-human serum albumin colloid and identification of one or more draining lymphocenters that included the SLN/SLNs using a gamma camera. The day of surgery, methylene blue was also injected peritumorally in four sites. Dogs were then moved to the operating theater. All tumors underwent wide-margin surgery (2–3 cm lateral margins and at least one deep fascial plane). For the intraoperative SLN detection, a hand-held gamma probe (Crystal probe SG04, Crystal Photonic GmbH, Berlin, Germany) was used to guide the excision of nodes with a radioactive count (RC) of at least twice the RC of a distant body region (background count). The SLN RC was checked ex vivo and further nodes belonging to the same lymphocenter were excised if the RC was equal to or greater than 10% of the RC of the hottest SLN removed. Any visible blue nodes were also excised and were identified as SLNs regardless of the RC.

After resection, MCT and SLNs were fixed in 10% neutral buffered formalin. All samples were routinely processed for histology and microtomic sections were stained with hematoxylin and eosin. SLNs were additionally stained with Giemsa. The primary tumor was histologically evaluated to obtain diagnosis of cutaneous or subcutaneous MCT, Patnaik and Kiupel’s histological gradings (for cutaneous MCT) [13,14], and margins status. To standardize the histological evaluation of the SLN, each lymph node was cut longitudinally at the level of hilus and multiple slices (1.5 mm thick) were obtained from each half of the lymph node when thicker than 3 mm (minor axis).

### 2.3. Recording Data

Recorded data were signalment (breed, age, sex, weight), MCT anatomic site (divided as previously reported in head and neck, trunk, limb, inguinal region, digit, and tail) [3], MCT size (maximum diameter measured with caliper), MCT presentation (first vs. recurrence), MCT ulceration, number of SLNs removed, number of lymphocenters evaluated, MCT location (cutaneous vs. subcutaneous), cutaneous MCT grade (based on both Patnaik et al., 1984 and Kiupel et al., 2011) [13,14], histological margin evaluation (tumor-free vs. infiltrated), histologic node status (HN) of the SLN/SLNs removed (according to Weishaar et al., 2014: HN0—non metastatic; HN1—pre metastatic; HN2—early metastasis; HN3—overt metastasis) [15]. Histologic growth pattern (circumscribed, infiltrative, or combined), mitotic index (0, ≤4, and >4), and presence or absence of multinucleation were also reported for subcutaneous MCT [16].

### 2.4. Statistical Methods

In case of MCT drained by multiple SLNs, the higher HN was assigned for statistical purposes.

#### 2.4.1. Association between HN and MCT Clinicopathological Variables

The association between HN and MCT clinical and histopathological variables was evaluated by generalized linear models with binomial error. Since the prevalence of HN was the clinically useful measure to evaluate the strength of association, the log-binomial model was preferred to logistic model for categorical variables [17]. For variables measured on continuous scale, log-binomial model fails to converge, thus estimated regression coefficients cannot be considered. For this reason, a generalized linear model with Poisson error with a robust variance–covariance matrix estimation was used [18].

Two separate analyses were performed: the first for HN0-1 vs. HN2-3 and the second for HN0-HN1-HN2 vs. HN3. Model response was the HN category, coded as 0 if HN0-1 and 1 if HN2-3 for the first analysis, and coded as 0 if HN0-HN1-HN2 and 1 if HN3 for the second analysis. Explanatory variables were both categorical and continuous. Continuous variables (size, number of SLN, number of lymphocenters) were included into the model in their original measurement scale. Categorical variables (anatomic site, ulceration, Patnaik grade, and cutaneous/subcutaneous location) were codified as dummy variables, thus for a categorical variable with K categories, K-1 dummy variables were included into the regression model and one of the categories was considered as the reference one. The variable “anatomic site” was categorized in 2 groups: sites historically associated with worse prognosis (head and neck, genital (including inguinal, scrotal, perivulvar and perineal) and digit) vs. sites historically associated with better prognosis (lateral thorax and abdomen, and limb, excluding digits) [19]. Longest tumor diameter was evaluated in its original continuous measurement scale (1 cm increase) and also considered as a categorical variable, coded as 0 if <3 cm and 1 if ≥3 cm [8,20]. Firstly, univariate analysis was performed for each of the above-mentioned variables. Results of the regression model were reported as prevalence ratio (PR) with corresponding 95% confidence intervals. The prevalence is the proportion of subjects with HN2-3 (or HN3). For each categorical variable with K categories, K-1 prevalence ratios are reported, each one representing the ratio between the prevalence for the category and the prevalence for the reference category. For continuous variables, PR is the ratio between the prevalence of HN2-3 (or HN3) for a unit increase of the variable. In the absence of association between a variable and HN, PR is expected to be 1. The null hypothesis of PR = 1 versus alternative hypothesis PR ≠ 1 was tested by Wald statistics. 

Multivariable analysis was performed following the “events per variable” (EPV) rule [21]. Accordingly, it was possible to consider a model with 3 variables for HN0-1 vs. HN2-3, and no multivariable analysis was possible for HN0-1-2 vs. HN3.

#### 2.4.2. Cluster Analysis and Association with HN

To investigate the joint association between the seven clinico-pathological variables (explanatory variables) and HN (model response), the appropriate method should have been a multivariable analysis including all the seven variables as predictors. To allow the mentioned analysis, a case series with at least 70 HN3 should have been available. Hence, a dimension reduction technique was applied as an alternative to summarize the patterns of association of the variables in a novel single variable having a small number of categories. The novel variable was obtained by processing all the explanatory variables (except MCT location, because of the high correlation with MCT histological grade) by cluster analysis (agglomerative hierarchical clustering on results from a factor analysis) [22] in such a way that dogs classified in the same category (cluster) were more similar for overall explanatory variables than dogs classified in other categories. The overall association between clusters and HN was evaluated by Fisher exact test. When the number of HN2-3 (or HN3) was adequate, the association was also evaluated by including clusters as explanatory variable in a generalized regression model with HN as the response variable.

To provide a clinical interpretation of the association between clusters and HN, a concise description of the explanatory variables which mainly characterized each cluster was obtained with a classification tree procedure [23] The aim was to build a criterion which classified the clusters as a function of the six explanatory variables. The procedure consisted of partitioning data repeatedly in groups which are increasingly homogeneous for cluster classification. Each partition was determined by the explanatory variable which best separated two groups to increase homogeneity within each group. The partition was stopped when each final group was composed by individuals classified in the same cluster. A further advantage of the tree classification procedure is to visualize the hierarchical contribution of each variable to the clusters.

#### 2.4.3. Predictive Impact of Variables on HN

A statistically significant prevalence ratio for a clinical pathological variable provides only partial information about the predictive impact of the variable. In fact, statistical significance does not imply a significant discriminant ability, the latter being related to the probability of correctly classifying the HN of each patient as a function of the values of the variable. The ability of the variables to discriminate between HN0-1 and HN2-3 (or between HN0-1-2 and HN3) was evaluated by calculating the area under ROC curve (AUC). For putative prognostic factors AUC values range from 0.5 to 1, where 1 indicates perfect discrimination ability (i.e., when each MCT with HN2-3 (or HN3) has the worse category of the clinico-pathological variable than each MCT with HN0-1 (or HN0-1-2)), and 0.5 indicates no discriminant ability (i.e., to use variable category like a flip coin). AUC values from −1 to <0.5 can be obtained only if MCT with HN2-3 (or HN3) have the better category of the clinico-pathological variable than patients with HN0-1 (or HN0-1-2). The null hypothesis AUC = 0.5 versus alternative hypothesis AUC > 0.5 was tested by Mann–Whitney U-test [24]. As an aid to evaluate the strength of discrimination ability, the following criterion was used: below 0.55 negligible; 0.55–0.63 small; 0.64–0.70 moderate; 0.71 and above strong [25]. 

All analyses were performed with a software package (R-Software; www.r-project.org, accessed on 10 May 2021), packages logbin (https://cran.r-project.org/web/packages/logbin/, accessed on 10 May 2021), sandwich (https://cran.r-project.org/web/packages/sandwich/, accessed on 10 May 2021), FactoMineR (https://cran.r-project.org/web/packages/FactoMineR/, accessed on 10 May 2021), ROCit (https://cran.r-project.org/web/packages/ROCit, accessed on 10 May 2021), and Verification (https://cran.r-project.org/web/packages/verification, accessed on 10 May 2021). A *p* ≤ 0.05 was considered significant.

## 3. Results

### 3.1. Sample Population

Fifty-three dogs were included in the study for a total of 66 integumentary MCT excised with their respective SLN/SLNs. Forty-four dogs had a single MCT, and 9 dogs had multiple MCT (simultaneous or not). Breeds were distributed as follows: 12 (22.7%) mixed-breeds, 10 (18.8%) Labrador Retrievers, 5 (9.4%) English Setter, 4 (7.5%) Golden Retrievers, 4 (7.5%) American Staffordshire, 3 (5.7%) Pug, 3 (5.7%) Boxers, 2 (3.7%) Maltese, 1 (1.9%) each of Dogo Argentine, Greater Swiss Mountain Dog, Bull Terrier, Beagle, Bracco Italiano, Tosa, Dachshund, Chihuahua, Weimaraner and Wire Fox Terrier. There were 22 (41.5%) intact males, 21 (39.6%) spayed females, 7 (13.2%) neutered males, and 3 (5.7%) intact females. Mean and median age at presentation were 7.6 and 8 years (range, 1–14 years), respectively. Mean and median weight were 25.8 and 27.5 kg (range, 3–62 kg), respectively. 

The 66 tumors were located as follows: 28 on the trunk, 18 on limbs, 9 on the head and neck, 8 on the inguinal region, 2 on the digits and 1 on the tail. Based on previous literature [19], 47 MCT occurred at a favorable prognostic site, while 19 occurred at a site associated with negative prognosis. All but 1 MCT were at first presentation. Tumor mean and median maximum diameter were, respectively 1.9 and 1.5 cm (range, 0.3–7.60 cm). Forty-nine MCT were smaller than 3 cm, and 17 were larger or equal to 3 cm. Ten out of 66 (15.1%) MCT were ulcerated. 

Histologically, 50 MCT were cutaneous, and 16 MCT were subcutaneous. All cutaneous MCT were Kiupel-low-grade, of which 12 were Patnaik-grade-I and 38 were Patnaik-grade-II. Eight subcutaneous MCT had an infiltrative growth pattern, 6 had a combined growth pattern and 2 were circumscribed. The mitotic index was 0 in 11 subcutaneous MCT, ≤4 in 4 subcutaneous MCT, and >4 in 1 subcutaneous MCT. Two subcutaneous MCT presented multinucleation, in the others the multinucleation was absent. Excisional margins were infiltrated in 4 cases.

A total of 115 SLNs were removed. A single SLN was found in 35 cases, 2 SLNs were removed in 17 cases, 3 SLNs were removed in 10 cases, and 4 SLNs were removed in 4 cases. The SLNs belonged to the same lymphocenter in 53 MCTs, and to two different lymphocenters in 13 MCTs. Histologically, 47 SLNs were classified as HN0, 16 as HN1, 42 as HN2 and 10 as HN3. Considering the maximum HN value for each MCT, 21 MCT had a HN0 SLN, 9 MCT had HN1 SLN, 29 MCT had a HN2 SLN, and 7 MCT had a HN3 SLN.

### 3.2. Association between HN Category and MCT Clinicopathological Variables

#### 3.2.1. HN0-1 vs. HN2-3

Results of univariate analysis are summarized in Table 1. Only size of the MCT and number of SNL were associated with SLN status: dogs with MCT larger than or equal to 3 cm had a higher prevalence of HN2-3 SLN compared to dogs with smaller tumors (PR = 1.629, *p* = 0.016). In addition, even when size was evaluated in its continuous scale the prevalence of HN2-3 SLN increased proportionally with each cm increase in tumor diameter (PR = 1.161, *p* = 0.002). The prevalence of HN2-3 also increased with the increasing number of SNL (PR = 1.240, *p* = 0.031). Although the AUC was significantly greater than 0.5 for tumor size (*p* = 0.019, *p* = 0.044), number of SNL (*p* = 0.021) and Patnaik grading systems (*p* = 0.036), the ability of the three variables to discriminate MCT with HN0-1 SLN from MCT HN 2-3 SLN was small (AUC < 0.64).

Since 36 HN2-3 SLN were available, a maximum of 3 variables could be included in the multivariable regression model. According to clinical relevance, the following variables were selected to evaluate their joint association with HN0-1 vs. HN2-3: size (continuous scale), number of SNL (continuous scale) and MCT location (cutaneous vs. subcutaneous) (Table 2). The ability of the model to discriminate between HN0-1 and HN2-3 patients was statistically significant (*p* = 0.004) but moderate (AUC = 0.69). When location of MCT was removed from the model, the other two variables retained the statistical significance and a negligible decrease was obtained from the model discriminant ability (AUC = 0.68). 

#### 3.2.2. HN0-1-2 vs. HN3

Results of univariate analysis are summarized in Table 3. Tumor size was confirmed as a prognostic variable related to the risk of a HN3 SLN both for the 3 cm cut-off and for 1 cm increase (PR = 7.206 *p* = 0.012 and PR = 1.648 *p* < 0.001). The ability of these variables to discriminate between HN0-1-2 and HN3 SLNs was significant (*p* = 0.002 and 0.006) and strong (AUC > 0.71). In addition to size, subcutaneous MCTs were also associated with the presence of at least one HN3 SLN (PR = 4.167 *p* = 0.044), with a significant (*p* = 0.018) and moderate discriminant ability (AUC = 0.68).

A significant strong discrimination ability was also found for Patnaik grading systems (AUC = 0.70 *p* = 0.026). 

### 3.3. Cluster Analysis and Association with HN

To evaluate the association between HN and the whole set of clinical and pathological variables reported in Table 1 and Table 3, dogs were grouped in clusters determined by the similarity of their clinical-pathological characteristics according to site (favorable vs. negative prognosis), size (≥3 cm vs. <3 cm), ulceration (yes vs. no), SLN number (1 vs. >1), SLN lymphocenter (1 vs. 2), MCT histological grade (subcutaneous, Patnaik I, Patnaik II). 

**Figure 1:**
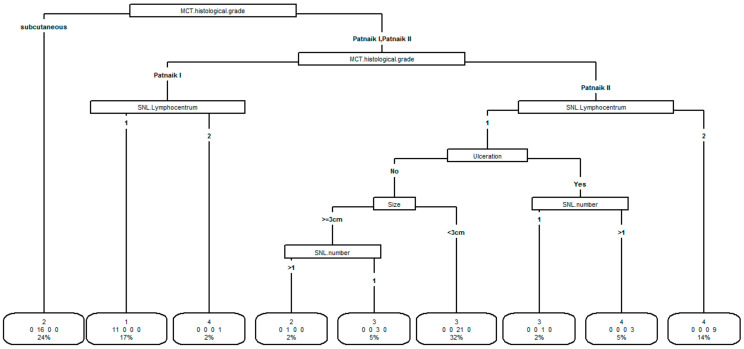
Results of the tree procedure to classify clusters according to the variables site (favorable vs. negative prognosis), size (≥3 cm vs. <3 cm), ulceration (yes vs. no), SLN number (1 vs. >1), SLN lymphocenter (1 vs. 2), MCT histological grade (subcutaneous, Patnaik I, Patnaik II).

Four clusters were identified (cluster 1:11 patients, cluster 2:17 patients, cluster 3:25 patients, cluster 4:13 patients). The tree classification procedure of clusters is reported in Figure 1.

The importance of the variables to identify clusters is represented in hierarchical order. The most important was MCT histological grade. Each main partition was further subdivided in sub-partitions having each one a more homogeneous cluster composition. The procedure ended with the final classification in which each group is made by subjects classified in the same cluster. All variables were used except tumor site which is not identified as useful to identify clusters after the previous variables were included.

In the boxes of final classification is reported the group cluster, the number of patients in the group, and the percentage of the patient in the group over the total of patients. 

The variable which mainly characterized the clusters was MCT histological grade, followed by SNL lymphocentrum, ulceration, size and number of SNL. The variable “tumor site” did not appear to be useful in classifying clusters after entering the above-mentioned variables in the model.

All the 11 dogs in cluster 1 were mainly characterized by Patnaik I MCT and one SNL lymphocenter. Dogs in cluster 2 were mainly characterized (16/17 dogs) by subcutaneous MCT. Dogs in cluster 3 were mainly characterized (21/25 patients) by Patnaik II MCT and one SLN lymphocenter, absence of ulceration, and tumor size <3 cm. In cluster 4, dogs were mainly characterized (9/13 dogs) by Patnaik II MCT and two SLN lymphocenters. 

The overall association between clusters and HN (classified as HN0-1 vs. HN2-3) was statistically significant (Fisher exact test *p*-value = 0.0049) as well as the ability of clusters to discriminate subjects with HN0-1 and HN2-3 (AUC = 0.68, 95% confidence interval 0.56–0.8, *p*-value = 0.004) (Table 4). 

The highest prevalence of HN3 SLNs was observed in cluster 2 (4/17, 25.8%). In cluster 4 the prevalence was 15.4% (2/13) and in cluster 1 it was 9.1% (1/11). None of the 25 dogs in cluster 3 had a HN3 SLN. Overall, the association between HN (classified as HN0-1-2 vs. HN3) and clusters was statistically significant (Fisher exact test *p* = 0.047). Due to the low number of HN3 SLN and the absence of HN3 SLN in cluster 3, it was not possible to estimate PR (prevalence ratio; the ratio between the prevalence of HN3 in each cluster and prevalence in cluster 3) by a regression model. The ability of cluster classification to discriminate between HN0-1-2 and HN3 was strong (AUC = 0.78), suggesting a potential usefulness of clusters to identify dogs with HN3 SLN.

## 4. Discussion

The increase of knowledge in small animal oncology has drawn interest towards the early detection of metastatic disease even in the absence of clinically relevant alterations. This concept has been applied, for instance, in dogs and cats admitted to second-level diagnostic imaging and to ultrasound-guided cytological sampling of macroscopically normal liver and spleen [8,10,26,27,28]. Unfortunately, patterns of imaging features and fine needle aspiration are insufficient to determine the status of the lymphatic basin in dogs with MCT with high repeatability [1,2,5]. Thus, histology on the excised lymph nodes remains a cornerstone to assess their metastatic involvement. Although this strategy enjoys the advantages of an early diagnosis of lymph node metastasis and allows for correct prognostication and therapeutic choices in a portion of cases, it could involve an unnecessary surgical procedure for those dogs with non-metastatic SLNs, and additional costs for the owners. These considerations have led to question whether SLN mapping and biopsy should always be performed or could be avoided in selected dogs.

Firstly, our study confirms the presence of early (HN2) and overt (HN3) metastasis in 54% of the sample population, even in case of subcutaneous and low-grade cutaneous MCT in dogs. In contrast, the remaining 46% of dogs were admitted to the surgical procedure even if SLNs were ultimately non metastatic. 

Based on our results, tumor size was associated with SLN metastasis. In 2015, a study conducted on more than 300 dogs with cutaneous MCT found a correlation between tumor size and nodal metastasis at admission, although RLN biopsy was performed in 50 cases only [20]. The overall rate of nodal metastasis in that study was lower than in the present study (18.1%) and nodal metastases were not categorized following Weishaar’s system [20]. In another study evaluating the RLN, tumors larger than 3 cm had a significantly higher risk of HN1-2-3 RLN; although tumor size lost its significance when HN2-3 RLN were considered jointly, the risk ratio was 1.40 [3]. This contradictory result may have been due to a discrepancy between the RLN and the actual draining SLN, according to the reportedly low correspondence between clinically expected RLN and SLN [4,5,6,7]. More recently, a significant correlation between MCT size (1 cm increase) and HN2-HN3 SLN was found in 35 cases, with SLN metastases occurring only in MCT greater than 26 mm [5]. In the present study, MCT size was associated with early and overt metastasis (HN2-3) and, by a greater extent, with overt metastases (HN3) analyzed alone. The risk of having HN2-HN3 (or HN3) increased for each cm increase in MCT diameter and for MCT larger than 3 cm. It may thus be argued that for tumors smaller than 3 cm, SLN mapping and biopsy could be avoided; however, it should be underscored that the discriminant ability of tumor size remains moderate. According to the current veterinary literature, the removal of HN2 lymph nodes has a potential therapeutic effect and should thus be performed anyway [29,30]. Further studies comparing the outcome of dogs with tumors smaller than 3 cm admitted or not to SLN biopsy are needed to confirm the unnecessary nature of the procedure in these cases. Conversely, the strong association that we report between tumor dimension and HN3 SLN when considering the HN3 category alone suggests that SLN mapping and biopsy should always be performed for MCT bigger than 3 cm without clinically evident nodal alterations. 

The number of SLN detected for each MCT was also associated with HN2-3. In human breast cancer the detection of a greater number of SLNs is often correlated with larger tumor size [31]. However, in the present sample population of canine MCT, the prevalence ratio of this variable remained statistically significant even in multivariate analysis when adjusted for tumor size, leading to hypothesize an absence of correlation between the number of SLN and size. A hypothesis is that the initiation of the metastatic process and nodal involvement could activate additional lymphatic networks, thus increasing the number of SLN detected. Further studies should elucidate the real significance of this finding, taking into account the difference in the number of SLN detected with different mapping techniques as well [32]. Moreover, the exact number of SLN to be removed is a post-operative variable not available before admitting the dog to the entire mapping and biopsy procedure. However, this data, if further confirmed, could help clinicians to prepare owners to a possible nodal metastatic status of their dog while waiting for the confirmation of the histopathology. 

Surprisingly, subcutaneous location was significantly associated with the presence of overt metastasis (HN3). Subcutaneous MCT represents a unique subset of tumors to which the histological grading systems do not apply. Few studies have focused on subcutaneous MCT and reported an overall good prognosis with metastatic rate ranging from 2 to 6% and prolonged survival (>1000 days) [16,33,34]. In those studies, data on nodal metastasis at admission were absent or biased by the absence of lymph node samples (either cytology or histology) in many of the included cases [16,33,34]. In two recent papers, 60% of subcutaneous MCT presented a ≥ HN2 SLN, although no association between cutaneous versus subcutaneous location and presence of nodal metastases was found [4,5]. In the present study, 68% of the 16 subcutaneous MCT had an early (HN2) or overt (HN3) SLN metastasis, confirming the high prevalence of nodal metastasis in this specific MCT subset. A moderate discriminant ability for the risk of having an HN3 SLN was found for subcutaneous versus cutaneous MCT, and the highest prevalence of HN3 SLN occurred in cluster 2 that included all dogs with subcutaneous MCT. Based on these results, the mapping and biopsy of SLNs should be strongly suggested in presence of subcutaneous MCT. However, it should be noted that the clinical identification of subcutaneous versus cutaneous MCT is not always straightforward, and some subcutaneous tumor could thus be overlooked if considered cutaneous during the pre-operative clinical examination [4]. Other histological characteristics have been reported to impact the prognosis of subcutaneous MCT, such as mitotic index, multinucleation, infiltrative pattern, Ki-67, and AgNOR [16,33,34]. In the present paper, mitotic index, multinucleation, and growth pattern were available, but the small number of subcutaneous cases did not permit to include this data in the statistical models. Further studies focusing on subcutaneous MCT only are thus needed to define a possible nodal metastatic risk stratification, after complete staging and SLN biopsy, by histological and immunohistochemical evaluation.

For cutaneous tumors, the Patnaik grading system did not associate with the SLN status, as reported in a recent study [5]. This result is probably due to the absence of Patnaik grade III–Kiupel high grade and Patnaik grade II–Kiupel high grade tumors in the present study. In the retrospective paper by Stefanello et al. (2015), 46.5% of Patnaik grade III and 29.7% of Kiupel high grade MCT had nodal metastasis at admission. In that study, the RLN was evaluated cytologically and/or histologically only in 18% of the dogs, although the clinical evaluation of the RLN was reported in all animals [20]. Therefore, the cytological and histological samples were probably reserved to clinically abnormal RLNs in many cases [20]. In Fournier’s study (2020), 10% of the SLN evaluated were enlarged at palpation, although it is not reported if they drained the four included high-grade tumors [5]. On the contrary, only dogs with non-palpable/normal sized RLN were admitted to the SLN mapping and biopsy in the present paper, thus probably influencing the absence of high-grade tumors. In both the above-mentioned papers as well as and in the present study, Patnaik grade II and grade I MCT did not have a different risk of nodal metastasis [5,20]. These results bring to the conclusion that variables other than Patnaik histological grade should be taken into account to define such risk in dogs with low-grade MCT. In addition, this result suggests that dogs who underwent excision of a low-grade MCT without SLN lymphadenectomy should be as well followed-up for nodal alteration.

A multivariate model including all the variables as predictors would have been the appropriate method to evaluate the joint interaction between multiple variables. As reported in the material and methods section, a case series with at least 70 MCT with HN3 SLN should have been available to allow the inclusion in the aforementioned analysis of all the seven clinicopathological variables examined in the present study. As an alternative, patterns of association of the seven variables were summarized in a single variable (cluster) obtained by classifying dogs in a small number of groups, in such a way that dogs classified in the same group were more similar for overall clinicopathological characteristics than dogs classified in other groups. When clusters were associated with HN0-1 vs. HN2-3, the association was significant but only a moderate ability of discrimination was found. However, the ability increased when the association was tested for HN0-1-2 vs. HN3. These results may suggest a potential usefulness of clusters in the identification of dogs with overt metastatic SLN. However, the precision of the estimates for the association of the variables with HN0-1-2 vs. HN3, both in univariate and clusters analyses, was low (wide confidence intervals) because of the low number of dogs with HN3 SLN, and further studies are needed. 

The final decision to perform the sentinel lymph node mapping and biopsy should take into account the association with the nodal staging (as tested in the present study) and the benefit of such procedures on the oncological outcome. As previously cited, recent papers on RLN reported a possible therapeutic effect of lymphadenectomy in the presence of early metastasis in canine MCT, although data on SLN are lacking [29,30]. In the study population, only 3 out of 53 dogs had an MCT relapse at a median follow-up time of 455 days (range, 15–1305 days) (data not shown). Hence a prognostic analysis on tumor progression and survival was not performed. Indeed, statistical analysis on such a low number of events would have led to obtaining unreliable statistical results. The low number of relapses could be hypothetically linked to the less aggressive behavior of the included MCT despite the presence of a high percentage of nodal metastasis; another potential explanation is the possible therapeutic effect of lymphadenectomy for SLN as well, and the need for a longer follow-up time for dogs with a less aggressive tumor in which surgery drastically decreased the burden of the disease. Further studies with a larger number of dogs and a longer follow-up time, and possibly comparing dogs admitted and not admitted to SLN biopsy, should be focused on prognostic data.

## 5. Conclusions

Although literature reported only few and self-limiting side effects of SLN biopsy in dogs [5,6], the mapping and removal of SLN involves additional anesthetic and diagnostic procedure, as well as additional cost for the owner. Based on the results of the present paper, tumor size in low-grade and subcutaneous MCT is associated with the risk of having HN2-HN3 SLN; however, considering the small to moderate ability of discrimination in distinguished HN2-HN3 from HN0-HN1, further studies should confirm this data before excluding dogs with smaller tumors from the nodal staging procedure. The high prevalence and the strong correlation between MCT equal or bigger than 3 cm, subcutaneous MCT and the presence of HN3 forces the solid suggestion to map and biopsy SLN in these cases.

## Figures and Tables

**Table 1:** Association between HN (HN0-1 vs. HN2-3) and MCT clinicopathological variables. Results of univariate analysis with log-binomial model (discrete variables) and Poisson model with robust variance (continuous variables).

Variable	PR	95% C.I.	*p*	AUC	95% C.I.	*p* *
Size						
3 cm vs. <3 cm	1.63	1.05–2.44	0.016	0.61	0.48–0.75	0.019
Increasing of 1 cm	1.16	1.06–1.28	0.002	0.62	0.49–0.76	0.044
Site						
Favorable vs. negative prognosis	0.83	0.44–1.33	0.479	0.54	0.40–0.68	0.232
Ulceration						
Yes vs. no	1.12	0.54–1.79	0.693	0.52	0.38–0.66	0.358
SLN number						
Increasing of 1 SLN	1.24	1.02–1.51	0.031	0.63	0.50–0.77	0.021
SLN lymphocenter						
Increasing of 1 lymphocenter	1.36	0.87–2.13	0.180	0.56	0.42–0.70	0.121
MCT location						
Cutaneous vs. subcutaneous	1.38	0.84–2.08	0.148	0.57	0.43–0.71	0.098
MCT histological grade						
Patnaik II vs. I	1.66	0.82–4.83	0.244			
subcutaneous vs. Patnaik I	2.06	0.97–6.05	0.101	0.62	0.48–0.75	0.036

Legend: PR = prevalence ratio, the ratio between the proportion of HN 2-3 in the two categories of the variable; C.I. 95% confidence interval; *p* = *p*-value of Wald test: null hypothesis PR = 1 vs. alternative hypothesis PR ≠ 1; AUC = area under ROC curve to measure discriminant ability of the variable (0.5 = no discrimination; > 0.5 and below 0.55 negligible; 0.55–0.63 small; 0.64–0.70 moderate; 0.71 and above strong), *p* * = *p*-value for Mann–Whitney U test: null hypothesis AUC = 0.5 versus alternative hypothesis AUC > 0.5.

**Table 2:** Association between HN (HN0-1 vs. HN2-3) and MCT clinicopathological variables. Results of multivariable analysis: Poisson model with robust variance.

Variable	PR	95% C.I.	*p*	AUC	95% C.I.	*p* *
Model with the three variables				0.69	0.57–0.82	0.004
Size						
Increasing of 1 cm	1.14	1.02–1.28	0.023			
SLN number						
Increasing of 1 SLN	1.22	1.01–1.47	0.044			
MCT location						
Cutaneous vs. subcutaneous	1.09	0.67–1.78	0.717			

Legend: PR = prevalence ratio, the ratio between the proportion of HN2-3 in the two categories of the variable; C.I. 95% confidence interval; *p* = *p*-value of Wald test: null hypothesis PR = 1 versus alternative hypothesis PR ≠ 1; AUC = area under ROC curve to measure discriminant ability of the model (0.5 = no discrimination; >0.5 and below 0.55 negligible; 0.56–0.63 small; 0.64–0.70 moderate; 0.71 and above strong); *p* * = *p*-value of Mann–Whitney U test: null hypothesis AUC = 0.5 versus alternative hypothesis AUC > 0.5.

**Table 3:** Association between HN (HN0-1-2 vs. HN3) and MCT clinicopathological variables. Results of univariate analysis with log-binomial model (discrete variables) and Poisson model with robust variance (continuous variables).

Variable	PR	95% C.I.	*p*	AUC	95% C.I.	*p* *
Size						
3 cm vs. <3 cm	7.21	1.73–47.21	0.012	0.76	0.54–0.97	0.002
Increasing of 1 cm	1.65	1.35–2.01	<0.001	0.74	0.58–0.99	0.006
Site						
Favorable vs. negative prognosis	0.99	0.15–4.18	0.989	0.5	0.27–0.73	0.500
Ulceration						
Yes vs. no	0.93	0.05–4.74	0.946	0.5	0.28–0.73	0.480
SLN number						
Increasing of 1 SLN	1.69	0.84–3.38	0.14	0.62	0.39–0.85	0.129
SLN lymphocentrum						
Increasing of 1 lymphocentrum	3.06	0.78–12.02	0.109	0.63	0.40–0.86	0.055
MCT location						
Cutaneous vs. subcutaneous	4.17	1.02–19.42	0.044	0.68	0.46–0.91	0.018
MCT histological grade						
Patnaik II vs. I	0.63	0.07–12.89	0.697			
Subcutaneous vs. Patnaik I	3.00	0.52–54.76	0.296	0.70	0.47–0.93	0.026

Legend: PR = prevalence ratio, the ratio between the proportion of HN3 in the two categories of the variable; C.I. 95% confidence interval for prevalence ratio; *p* = *p*-value of Wald test: null hypothesis PR = 1 versus alternative hypothesis PR ≠ 1; AUC = area under ROC curve to measure discriminant ability of the variable (0.5: no discriminant ability; >0.5 and below 0.55 negligible; 0.55–0.63 small; 0.64–0.70 moderate; 0.71 and above strong); *p* * = *p* value of Mann–Whitney U test: null hypothesis AUC = 0.5 versus alternative hypothesis AUC > 0.5.

**Table 4:** Association between clusters and HN2-3. Results of log-binomial model. Prevalence is the proportion of HN2-3 within cluster, PR is the ratio between the prevalence of HN2-3 in each cluster and prevalence in cluster 3 (considered as reference because of the most represented cluster).

Cluster (Number of MCT)	Prevalence of HN2-3	PR	95% C.I.	Z	*p*
1 (*n* = 11)	0.364	0.91	0.36–2.28	−0.20	0.84
2 (*n* = 17)	0.706	1.77	0.99–3.12	1.96	0.05
3 (*n* = 25)	0.400				
4 (*n* = 13)	0.769	1.92	1.09–3.38	2.27	0.02

Legend: C.I. confidence interval; Z = Wald statistics; *p* is the *p*-value of Wald test for the hypothesis PR = 1 versus PR ≠ 1.

## Data Availability

The row data supporting the conclusions of this article will be made available by the corresponding author upon reasonable request, without undue reservation.
